# Exebacase Is Active *In Vitro* in Pulmonary Surfactant and Is Efficacious Alone and Synergistic with Daptomycin in a Mouse Model of Lethal Staphylococcus aureus Lung Infection

**DOI:** 10.1128/AAC.02723-20

**Published:** 2021-08-17

**Authors:** Steven M. Swift, Karen Sauve, Cara Cassino, Raymond Schuch

**Affiliations:** a ContraFect Corporation, Yonkers, New York, USA

**Keywords:** *Staphylococcus aureus*, MRSA, exebacase, CF-301, lysin, synergy, direct lytic agent, pneumonia

## Abstract

Exebacase (CF-301) is a novel antistaphylococcal lysin (cell wall hydrolase) in phase 3 of clinical development for the treatment of Staphylococcus aureus bacteremia, including right-sided endocarditis, used in addition to standard-of-care antibiotics. In the current study, the potential for exebacase to treat S. aureus pneumonia was explored *in vitro* using bovine pulmonary surfactant (Survanta) and *in vivo* using a lethal murine pneumonia model. Exebacase was active against a set of methicillin-sensitive S. aureus (MSSA) and methicillin-resistant S. aureus (MRSA) strains, with an MIC_90_ of 2 μg/ml (*n* = 18 strains), in the presence of a surfactant concentration (7.5%) inhibitory to the antistaphylococcal antibiotic daptomycin, which is inactive in pulmonary environments due to specific inhibition by surfactant. In a rigorous test of the ability of exebacase to synergize with antistaphylococcal antibiotics, exebacase synergized with daptomycin in the presence of surfactant *in vitro*, resulting in daptomycin MIC reductions of up to 64-fold against 9 MRSA and 9 MSSA strains. Exebacase was also observed to facilitate the binding of daptomycin to S. aureus and the elimination of biofilm-like structures formed in the presence of surfactant. Exebacase (5 mg/kg of body weight 1 time every 24 h [q24h], administered intravenously for 3 days) was efficacious in a murine model of staphylococcal pneumonia, resulting in 50% survival, compared to 0% survival with the vehicle control; exebacase in addition to daptomycin (50 mg/kg q24h for 3 days) resulted in 70% survival, compared to 0% survival in the daptomycin-alone control group. Overall, exebacase is active in pulmonary environments and may be appropriate for development as a treatment for staphylococcal pneumonia.

## INTRODUCTION

Staphylococcus aureus colonizes the skin and mucosal surfaces of up to 60% of the adult human population on a permanent or intermittent basis (1, 2) and is associated with clinical manifestations ranging from mild skin and soft tissue infections to severe and life-threatening diseases such as bacteremia, endocarditis, osteomyelitis, and pneumonia (3). Notably, staphylococci are the most common cause of bacterial pneumonia, at 19.2 cases/100,000 population, and are associated with the highest case-fatality rate for bacterial pneumonia, at 15.6 deaths/100 cases, despite antibiotic intervention (4). In the setting of an influenza epidemic, secondary respiratory infections with S. aureus are associated with increased morbidity, particularly in at-risk groups such as the immunocompromised/immunosuppressed (5, 6). During the severe acute respiratory syndrome coronavirus (SARS-CoV) outbreak in 2003, up to 30% of patients were diagnosed with secondary bacterial infections (including S. aureus), and coinfection was positively associated with disease severity (7). In a recent multicenter study that included 476 coronavirus disease 2019 (COVID-19) patients, secondary bacterial infections were also significantly associated with outcome severity (8).

For a growing number of infection types, including staphylococcal pneumonia, treatment is confounded by antibiotic resistance (9–11), resulting in longer hospital stays, higher medical costs, and increased mortality. This issue of rising and globally disseminated antibiotic resistance has created a public health crisis, which requires the development of novel antimicrobial agents, including those with mechanisms of action differentiated from those of traditional antibiotics (12).

Direct lytic agents (DLAs), including lysins, are a new antimicrobial modality to address the unmet need arising from antibiotic resistance (13, 14). Lysins are recombinantly produced cell wall peptidoglycan hydrolytic enzymes that elicit rapid cell wall cleavage and concomitant osmotic lysis. Exebacase is an antistaphylococcal lysin with the following microbiological attributes: (i) rapid, targeted bactericidal activity; (ii) the ability to eradicate staphylococcal biofilms; (iii) synergy with antistaphylococcal antibiotics, including daptomycin (DAP) and vancomycin; (iv) a low propensity for the development of resistance; (v) no cross-resistance with antibiotics; (vi) the capacity to both suppress antibiotic resistance and “resensitize” antibiotic-resistant bacteria; and (vii) an extended *in vitro* and *in vivo* postantibiotic effect (15–20). Exebacase recently became the first lysin with published results from a phase 2 proof-of-concept clinical trial, which demonstrated 42.8% higher clinical responder rates with a single dose of exebacase used in addition to standard-of-care antibiotics (SOCAs) than with SOCAs alone for the treatment of methicillin-resistant S. aureus (MRSA) bacteremia, including endocarditis (21). Breakthrough therapy designation has been granted by the U.S. Food and Drug Administration (FDA) for exebacase, which is now also in phase 3 of development (22).

In the present study, the antistaphylococcal activity of exebacase was tested *in vitro* in the presence of bovine pulmonary surfactant and *in vivo* in a murine pneumonia model to explore the use of exebacase as a treatment for staphylococcal lung infections, including pneumonia. In a rigorous test of the ability of exebacase to synergize with antistaphylococcal antibiotics, the potency of exebacase in pulmonary environments was examined in addition to daptomycin, a lipopeptide antibiotic approved for use in treating S. aureus skin and soft tissue infections and bacteremia but which is ineffective in treating bronchoalveolar pneumonia because of selective sequestration by and inactivation in pulmonary surfactant (23). The capacity of exebacase to synergize with daptomycin in this proof-of-principle study would highlight the potency of exebacase in pulmonary environments and its promise as a treatment for staphylococcal lung infections.

## RESULTS

### Exebacase is active in bovine pulmonary surfactant.

**FIG 1:**
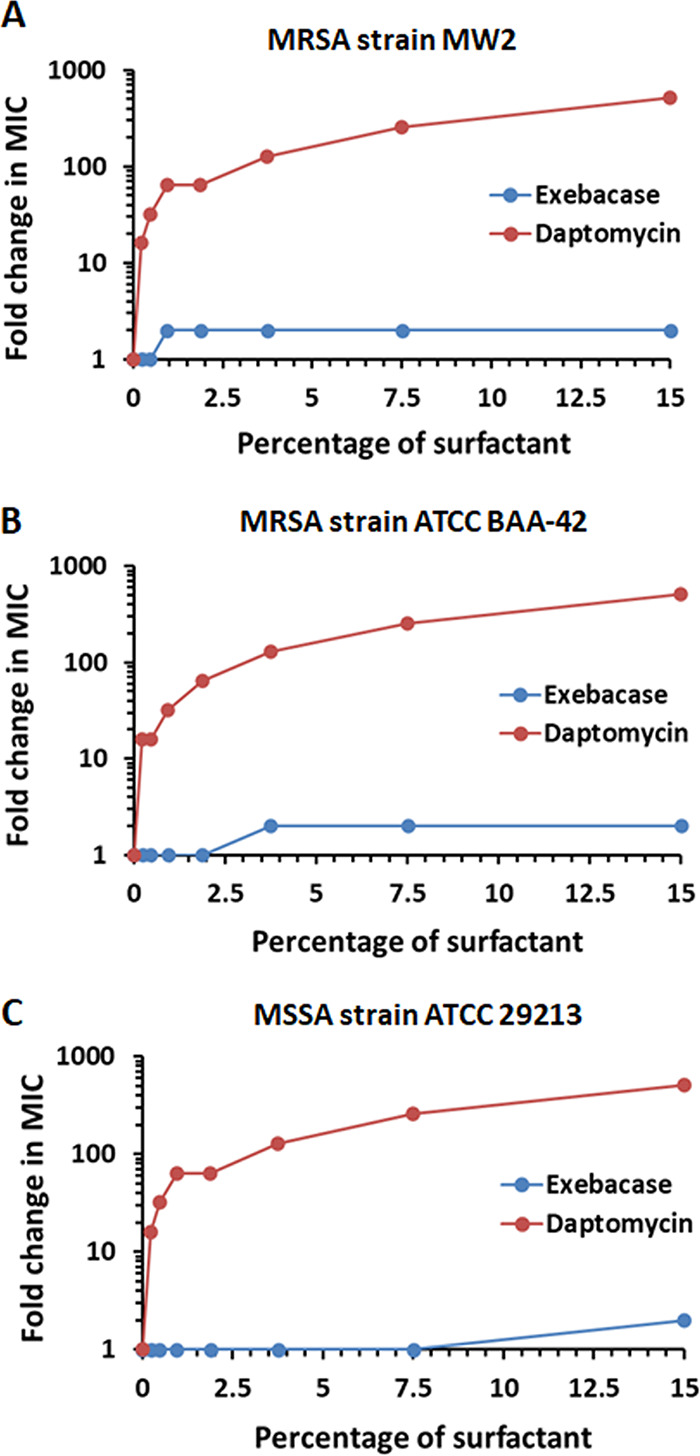
Exebacase is active in pulmonary surfactant (Survanta). MICs were determined by broth microdilution in CAMHB-HSD (for exebacase) or CAMHB supplemented with Ca^2+^ (for daptomycin) with the indicated concentrations of surfactant. Data are ratios of MICs with and without surfactant. Starting MIC values (i.e., in the absence of surfactant) for exebacase and DAP were 0.5 and 0.25 μg/ml, respectively, for S. aureus strains MW2 (A), ATCC BAA-42 (B), and ATCC 29213 (C).

The activity of exebacase was tested in medium supplemented with increasing amounts of bovine pulmonary surfactant (Survanta), a natural lung extract containing phospholipids, neutral lipids, fatty acids, and surfactant-associated proteins that is used to mimic the surface-tension-lowering properties of natural lung surfactant (24). As indicated in Fig. 1, exebacase was highly active against each of three S. aureus strains, over a range of surfactant concentrations from 1 to 15%. In contrast, daptomycin exhibited a 16- to 512-fold loss of activity for each S. aureus strain across the range of concentrations tested.

### Exebacase synergizes with daptomycin in 7.5% bovine pulmonary surfactant.

To inform concentration selection in checkerboard assays, single-agent MICs for exebacase and daptomycin were first determined by broth microdilution (BMD) against each of 9 methicillin-sensitive S. aureus (MSSA) and 9 MRSA strains in medium with 7.5% bovine pulmonary surfactant. Exebacase was highly active, with MICs ranging from 1 to 2 μg/ml for all strains tested (Table 1). Daptomycin MICs ranged from 16 to 128 μg/ml, consistent with inhibition of activity in the presence of surfactant. In contrast, MICs for exebacase and daptomycin in the absence of surfactant were each 0.5 to 1 μg/ml.

**TABLE 1:** Exebacase synergizes with daptomycin in checkerboard assays performed in the presence of 7.5% pulmonary surfactant (Survanta)^*a*^

Strain^*b*^	Exebacase			Daptomycin			FICI value	Type of interaction
	Single-agent MIC (μg/ml)	MIC in combination	Fold reduction in MIC	Single-agent MIC (μg/ml)	MIC in combination	Fold reduction in MIC		
MRSA								
BAA-42	1	0.25	4	64	4	16	0.313	Synergy
BAA-1747	2	0.5	4	64	1	64	0.266	Synergy
BAA-1688	1	0.25	4	64	2	32	0.281	Synergy
MW2	1	0.25	4	64	2	32	0.281	Synergy
NRS 265	1	0.25	4	64	1	64	0.266	Synergy
NRS 193	1	0.25	4	32	2	16	0.313	Synergy
NRS 255	1	0.25	4	16	1	16	0.313	Synergy
JMI 947	1	0.25	4	32	2	16	0.313	Synergy
JMI 3167	1	0.25	4	64	4	16	0.313	Synergy

MSSA								
ATCC 25923	1	0.25	4	64	2	32	0.281	Synergy
ATCC 29213	1	0.25	4	32	4	8	0.375	Synergy
ATCC 49521	1	0.25	4	64	2	32	0.281	Synergy
NRS 153	2	0.5	4	64	2	32	0.281	Synergy
NRS 131	1	0.25	4	64	2	32	0.281	Synergy
NRS 106	1	0.25	4	128	2	64	0.265	Synergy
JMI 316	1	0.25	4	64	1	64	0.265	Synergy
JMI 1040	1	0.25	4	64	2	32	0.281	Synergy
JMI 1173	2	0.5	4	32	1	32	0.281	Synergy

a For each isolate tested, MIC values are indicated for exebacase and daptomycin when tested alone (separately) in the presence of 7.5% pulmonary surfactant and when tested in addition to each other in the presence of 7.5% pulmonary surfactant. The fold reduction in the MIC is based on the decrease observed for each agent in the synergistic combination compared to the value obtained as a single agent.

b The bacterial strains are described in Table S1 in the supplemental material.

Based on the single-agent MICs determined as described above, exebacase was tested in addition to daptomycin against each of the 9 MSSA and 9 MRSA strains using a standard checkerboard assay format in medium with 7.5% pulmonary surfactant. Fractional inhibitory concentration index (FICI) values were assessed according to the following criteria: synergy at an FICI of ≤0.5, additivity at an FICI of >0.5 to ≤1, no interaction (indifference) at an FICI of >1 to ≤4, and antagonism at an FICI of >4. Exebacase synergized with daptomycin against each of the 18 strains tested (Table 1). Exebacase MICs were reduced 4-fold, while daptomycin MICs were reduced up to 64-fold. When used in addition to exebacase, the daptomycin MICs were at or near the susceptibility breakpoint of ≤1 μg/ml established by the Clinical and Laboratory Standards Institute (CLSI) (25).

### Exebacase promotes daptomycin binding to S. aureus in surfactant.

**FIG 2:**
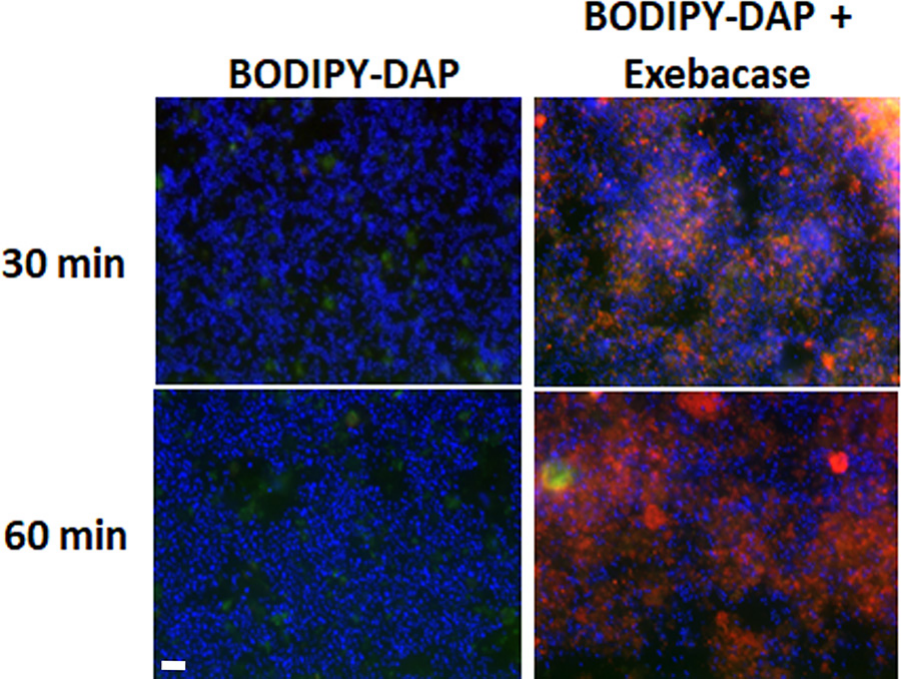
Exebacase promotes BODIPY-daptomycin (DAP) binding to MRSA strain MW2 in 7.5% surfactant. DAPI-labeled cells were treated with BODIPY-DAP (4 μg/ml; 1/8 MIC) in the presence and absence of exebacase (0.125 μg/ml; 1/8 MIC) for 30 and 60 min. Blue, DAPI labeling; red and green, BODIPY-DAP labeling. Magnification, ×2,000. Bar, 7 μm.

**FIG 3:**
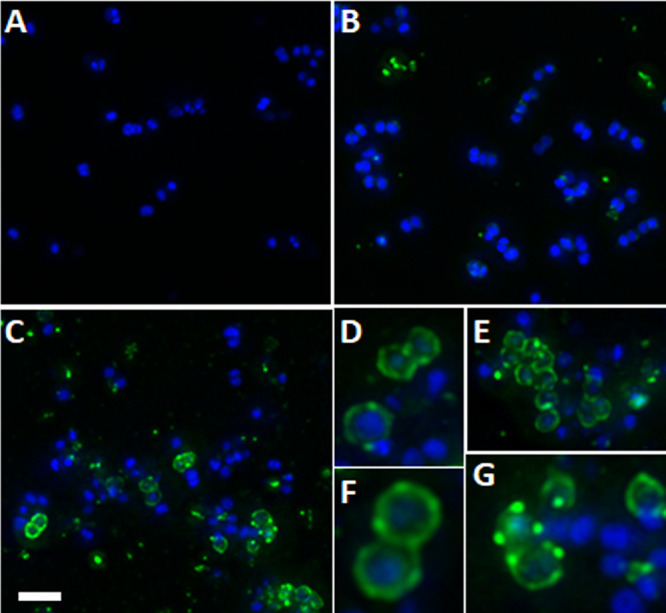
Exebacase promotes BODIPY-DAP binding to MRSA strain MW2 in 7.5% pulmonary surfactant. DAPI-labeled cells were treated for 30 min with either buffer (A), BODIPY-DAP (4 μg/ml; 1/8 MIC) (B), or BODIPY-DAP in addition to exebacase (0.125 μg/ml; 1/8 MIC) (C to G). Blue, DAPI labeling; green, BODIPY-DAP labeling. Bar, 2 μm.

The binding of BODIPY FL-labeled daptomycin (used at a sub-MIC) to MRSA strain MW2 was examined in 7.5% surfactant in the presence or absence sub-MIC exebacase, using both epifluorescence (Fig. 2) and confocal (Fig. 3) microscopy. Fluorescently labeled boron-dipyrromethene (BODIPY-FL)-labeled daptomycin normally fluoresces green when inserted into the bacterial membrane target but can have a red shift in fluorescence emission when the signal is very intense from a high probe density (26). In the presence of CF-301, BODIPY FL-labeled daptomycin stained S. aureus green or red within 30 min, whereas without CF-301, no staining was observed.

### Exebacase and daptomycin act synergistically against S. aureus biofilms in surfactant.

**FIG 4:**
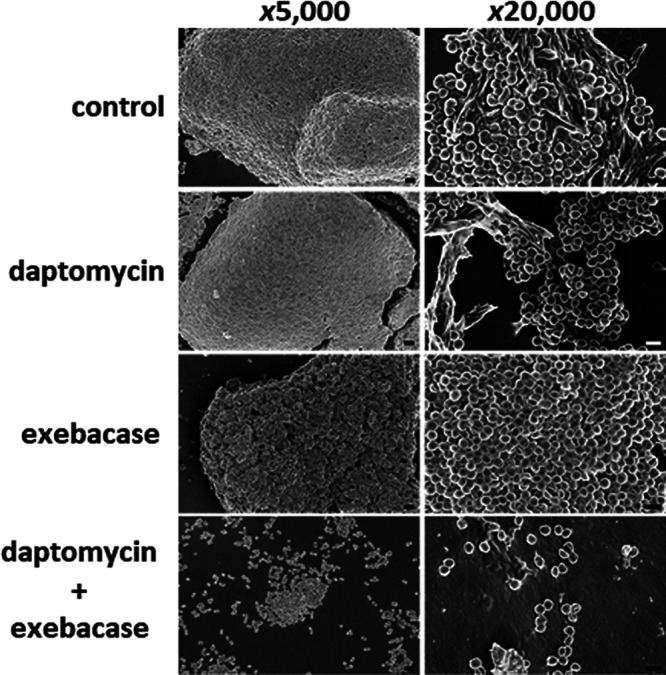
Exebacase and daptomycin act synergistically to reduce S. aureus biofilms formed in 7.5% surfactant. MRSA strain MW2 was treated for 20 min with either buffer (control), daptomycin (4 μg/ml; 1/8 MIC), exebacase (0.125 μg/ml; 1/8 MIC), or daptomycin in addition to exebacase. Bars, 2 μm (images at a magnification of ×5,000) and 1 μm (images at a magnification of ×20,000).

The activity of exebacase and/or daptomycin (each at sub-MICs) against biofilms formed by MRSA strain MW2 in 7.5% surfactant was examined by scanning electron microscopy (SEM). Treatments with daptomycin alone for 20 min resulted in densely packed formations of cells similar to those observed in the vehicle control (Fig. 4). While the 20-min treatment with sub-MIC exebacase alone was not disruptive, the biofilms were nonetheless porous and less dense than those observed with either the vehicle or daptomycin treatments. In contrast, exebacase in addition to daptomycin removed the biofilm, leaving only debris and scattered groups of individual bacteria after the 20-min treatment.

### Exebacase is efficacious in a murine pneumonia model.

**FIG 5:**
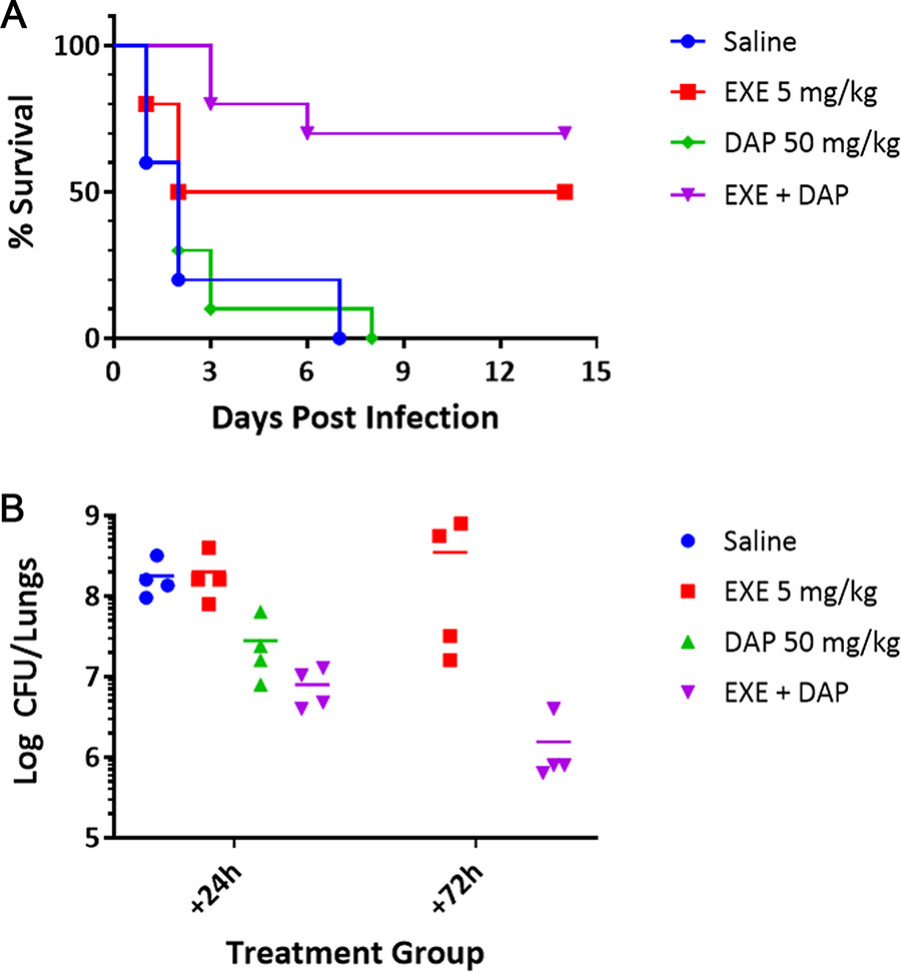
Efficacy study in a murine pneumonia model. (A) Survival of mice infected intranasally with 5 × 10^8^ CFU of MRSA strain ATCC BAA-42 and treated with either saline (vehicle control), exebacase (EXE) (i.v.), daptomycin (DAP) (s.c.), or exebacase in addition to DAP once daily for 3 days beginning 4 h after the start of infection (*n* = 10 mice/group). Data were analyzed by the log rank test (*P *< 0.05 for either exebacase alone or exebacase in addition to DAP versus DAP alone). (B) For surviving animals, bacteria were quantitated 24 h and 72 h after the start of infection for 4 mice/group. No survivors were observed at 72 h in the saline and DAP-alone groups. Data were analyzed by the Mann-Whitney U test (for the 24-h analysis, *P* < 0.05 for exebacase plus DAP versus the saline control and exebacase alone and for DAP alone versus the saline control; for the 72-h analysis, *P* < 0.5 for exebacase plus DAP versus exebacase alone).

A BALB/c mouse model of lethal pneumonia was established in which exebacase (5 mg/kg of body weight intravenously [i.v.]) and/or daptomycin (50 mg/kg subcutaneously [s.c.]) was administered for 3 days, 1 time every 24 h (q24h), starting 4 h after intranasal (i.n.) infection with 5 × 10^8^ CFU of MRSA strain ATCC BAA-42 (Fig. 5A). ATCC BAA-42 is a human respiratory isolate also known as strain HDE288 (36). At 14 days, mice treated with exebacase alone or in addition to daptomycin yielded 50% and 70% survival rates, respectively, whereas treatment with daptomycin alone or the vehicle yielded no survivors by 8 days. Exebacase alone and in addition to daptomycin was superior to either daptomycin alone or the vehicle control (*P* < 0.05 by a log rank test).

While survival was the primary endpoint in this proof-of-concept study, bacterial loads were also determined in the lungs of infected animals at the 1- and 3-day time points after the start of treatment. Only the exebacase plus daptomycin treatment group exhibited significant 1- and 2-log_10_ decreases compared to the starting bacterial inoculum at 1 and 3 days, respectively (Fig. 5B). Bacterial loads were not determined at the later time points up to 14 days.

## DISCUSSION

Novel antimicrobial agents with differentiated mechanisms of action compared to those of current and long-standing antibiotic classes are needed to confront the urgent unmet medical need resulting from drug- and multidrug-resistant bacteria, including S. aureus, a ubiquitous and versatile human pathogen. Direct lytic agents, in particular lysins, represent novel therapeutic modalities distinguished by a notably potent enzymatic mechanism of action (peptidoglycan hydrolysis) and bacteriolytic effect. The therapeutic potential of lysins is strongly supported by positive proof-of-concept data from a completed phase 2 clinical trial of exebacase and the initiation of the phase 3 DISRUPT trial to assess the efficacy and safety of exebacase used in addition to standard antibiotics in patients with S. aureus bacteremia, including right-sided endocarditis (21, 22).

In the current study, the potential for using exebacase as a treatment for another staphylococcal infection with a high unmet need (i.e., bronchopneumonia) was demonstrated. Exebacase exhibited potent activity and low MICs in growth media supplemented with bovine pulmonary surfactant, unlike daptomycin, which was inhibited by up 512-fold. The inactivity of daptomycin has been attributed to insertion into lipid aggregates within the surfactant, which then precludes insertion into the membrane of target Gram-positive bacteria (23); sequestration within and inhibition by the surfactant may explain the failure of daptomycin in clinical trials for community-acquired pneumonia. Conversely, the potent activity demonstrated for exebacase in bovine surfactant predicts a high level of activity for the enzyme in the presence of natural surfactant in pulmonary environments. Indeed, systemically administered exebacase, tested only at a 5-mg/kg dose, resulted in 50% survival among mice infected intranasally with an otherwise lethal dose of S. aureus. The efficacy of exebacase in the murine pneumonia model is consistent with the capacity to both penetrate the pulmonary environment and exert antistaphylococcal activity in the presence of a “natural” surfactant.

Synergy between exebacase and daptomycin was previously demonstrated in checkerboard and time-kill assays performed using cation-adjusted Mueller-Hinton broth supplemented with 25% horse serum and 0.5 mM dithiothreitol (CAMHB-HSD) albeit in the absence of surfactant (16). The synergy now demonstrated in the presence of surfactant is particularly compelling considering that daptomycin is inactive in surfactant in the absence of exebacase. By reducing the daptomycin MICs by up to 64-fold to nearly breakpoint values, synergy with exebacase “activated” the antibiotic. This activation was visualized and observed to be based on the enhanced binding of BODIPY-labeled daptomycin to the staphylococcal membrane target in the presence of exebacase. Synergy was furthermore observed to facilitate antibiofilm activity *in vitro* and enhanced efficacy (70% survival versus no survival in the vehicle control group) in the murine pneumonia model.

The mechanism by which exebacase promotes daptomycin binding and synergistic activity remains to be determined. Exebacase may diminish the sequestration of daptomycin within lipid aggregates in pulmonary surfactants, or the cell wall hydrolytic activity of exebacase may facilitate the access of nonaggregated antibiotics to the target membrane. While the pattern of BODIPY-DAP surface labeling observed in our study, which is defined by distinct foci of enhanced fluorescence, is similar to that previously reported (28, 29), it does not show distinct septal binding as has also been observed for daptomycin (30). Exebacase may thus displace the antibiotic from the division plane and favor accumulation at other sensitive sites.

Overall, we have provided evidence of the capacities of exebacase to kill S. aureus under *in vitro* conditions mimicking the respiratory environment and to confer a survival benefit *in vivo* in the lungs of experimentally infected mice. These findings support consideration for exebacase development as a novel treatment, with a differentiated mechanism of action, for staphylococcal pneumonia and other difficult-to-treat respiratory infections, including MRSA-related pulmonary exacerbations of cystic fibrosis. While exebacase has been shown to synergize with a wide range of antistaphylococcal antibiotics in checkerboard and time-kill assay formats (15, 16) and in rat and rabbit models of bacteremia and endocarditis (31), until now, the potential ability of exebacase to synergize with antibiotics in the setting of *in vivo* lung infections has not been demonstrated. Daptomycin, an antistaphylococcal antibiotic known to be inactive in the pulmonary environment, was specifically chosen as the “partner antibiotic” in this proof-of-principle study to provide the most rigorous test of the potential ability of exebacase to synergize with antistaphylococcal antibiotics. Based on the activity of exebacase observed in the current work, including the ability to synergize with daptomycin in the pulmonary environment, future work will include translational studies in higher-order species (e.g., the rabbit pulmonary infection model) testing the ability of exebacase to improve outcomes when used in addition to antistaphylococcal antibiotics commonly used clinically to treat pneumonia caused by S. aureus (e.g., vancomycin and linezolid, etc.) (32, 33).

## MATERIALS AND METHODS

### Bacterial strains and reagents.

Exebacase (CF-301) (>99% pure) was prepared by ContraFect Corporation (Yonkers, NY). Daptomycin was obtained from Sigma-Aldrich (St. Louis, MO). All S. aureus strains were obtained from the American Type Culture Collection (ATCC), BEI Resources (NRS), and JMI Laboratories, as indicated in Table S1 in the supplemental material. Frozen strains were revived on BBL Trypticase soy agar plates with 5% sheep blood (TSAB; Becton, Dickinson and Company [BD]) and incubated at 37°C overnight for single colonies. DAPI (4′,6-diamidino-2-phenylindole dihydrochloride) was obtained from Thermo Fisher Scientific. Other growth media included tryptic soy broth (TSB) from Hardy Diagnostics (VWR International), TSB with 0.2% d-glucose (TSBg), and BBL cation-adjusted Mueller-Hinton II broth (CAMHB; Becton, Dickinson and Company). Horse serum (donor herd, sterile filtered, and not heat inactivated) was obtained from Sigma-Aldrich. d-Glucose and dl-dithiothreitol (DTT) were obtained from Sigma-Aldrich. Survanta (Beractant) was obtained from Myonex Incorporated and is a modified bovine pulmonary surfactant consisting of 25 mg/ml phospholipids, 0.5 to 1.75 mg/ml triglycerides, 1.4 to 3.5 mg/ml free fatty acids, and <1.0 mg/ml total surfactant proteins.

### MIC assays.

Exebacase MICs were measured by broth microdilution (BMD) according to the CLSI M07-A11 methodology (34) and incorporating a CLSI-approved antimicrobial susceptibility testing (AST) medium comprised of CAMHB supplemented with horse serum (Sigma-Aldrich) and dl-dithiothreitol (Sigma-Aldrich) to final concentrations of 25% and 0.5 mM, respectively. This medium, referred to as CAMHB-HSD, was approved for use in exebacase AST (35) and is described in CLSI document M100-ed30 (25). Daptomycin MICs were determined using either standard AST medium (CAMHB supplemented with Ca^2+^ to 50 μg/ml) or CAMHD-HSD, as indicated; the equivalence of daptomycin MICs determined in CAMHB with Ca^2+^ and CAMHB-HSD was previously demonstrated (16). The impact of pulmonary surfactant on the activity of exebacase and daptomycin was tested as described above in medium supplemented with a range of Survanta concentrations (23).

### Checkerboard assays.

Fractional inhibitory concentration index (FICI) values were determined by broth microdilution on individual checkerboard panels in 96-well polystyrene microtiter plates containing exebacase diluted (2-fold) across the *y* axis and each antibiotic diluted (2-fold) across the *x* axis (16, 27). In this manner, exebacase was tested alone and in addition to daptomycin against 9 MSSA and 9 MRSA strains in CAMHB-HSD supplemented with 7.5% pulmonary surfactant (Survanta). Daptomycin MICs determined in CAMHB-HSD were previously demonstrated to be equivalent to MICs determined in CAMHB supplemented with Ca^2+^ (16); additionally, previously reported synergy studies using exebacase and daptomycin were performed in CAMHB-HSD (16). FICI values were assessed according to the following criteria: synergy at an FICI of ≤0.5, additivity at an FICI of >0.5 to ≤1, no interaction (indifference) at an FICI of >1 to ≤4, and antagonism at an FICI of >4.

### Epifluorescence and confocal microscopy.

The coupling of daptomycin to amine-reactive BODIPY FL-X succinimidyl ester was performed as described by the manufacturer (Thermo Fisher Scientific). BODIPY FL-labeled daptomycin was purified over a PD MiniTrap G-10 column (Cytiva), and activity was confirmed by an MIC assay by broth microdilution in CAMHB supplemented with Ca^2+^ to 50 μg/ml; the MIC of labeled daptomycin was equivalent to that of unlabeled material. For the epifluorescence and confocal microscopy studies, mid-log-phase cells (MRSA strain MW2) were washed and resuspended in 25 mM Tris (pH 7.2) with 50 μg/ml CaCl_2_ and 7.5% Survanta surfactant. The DAPI stain was next added according to the manufacturer’s protocol (Thermo Fisher), followed by BODIPY FL-daptomycin (4 μg/ml; 1/8 MIC) and/or exebacase (0.125 μg/ml; 1/8 MIC). After incubation for either 30 or 60 min at room temperature, cells were diluted, washed, and applied to the surface of 0.01% lysine-coated slides. Slides were mounted in 50% glycerol and 0.1% *p*-phenylenediamine in phosphate-buffered saline (PBS) (pH 8). Epifluorescence microscopy was performed using a Nikon Eclipse E400 microscope equipped with a Nikon 100×/1.25-numerical-aperture (NA) oil immersion lens and a Retiga EXi fast 1394 camera (QImaging). QCapture Pro version 5.1.1.14 software (QImaging) was used for image capture and processing. Confocal (deconvolution) microscopy was performed using a DeltaVision image restoration microscope (Applied Precision/Olympus) equipped with a CoolSnap QE cooled charge-coupled-device (CCD) camera (Photometrics). Images were captured using an Olympus 100×/1.40-NA plan apochromat oil immersion objective combined with a 1.5× Optovar magnification enhancer. The Z-stacks were taken at 0.15-min intervals. Images were deconvolved using SoftWoRx software (Applied Precision/DeltaVision), corrected for chromatic aberrations, and presented as maximum-intensity projections combining all relevant Z-sections.

### Scanning electron microscopy.

MRSA strain MW2 was grown overnight in CAMHB-HSD supplemented with 7.5% Survanta, and culture aliquots were washed and resuspended in 25 mM Tris (pH 7.2) (with 50 μg/ml CaCl_2_ and 7.5% surfactant) before treatment for 20 min with either buffer alone or buffer with daptomycin (4 μg/ml; 1/8 MIC) and/or exebacase (0.125 μg/ml; 1/8 MIC). The samples were postfixed in 1% osmium tetroxide, block stained with uranyl acetate, and processed for visualization using a Tecnai Spirit BT transmission electron microscope (FEI).

### Murine lung infection model.

Female BALB/c mice, 5 to 7 weeks of age, with body weights of 16 to 19.5 g (Jackson Laboratories) were used. Exponential-phase bacterial inocula (MRSA strain ATCC BAA-42) were generated by growing cells to an optical density at 600 nm (OD_600_) of 0.5, harvested, washed, and concentrated to 1.5 × 10^9^ to 2 × 10^9^ CFU/ml in sterile saline. Mice were anesthetized with intraperitoneal ketamine and xylazine and then infected intranasally (i.n.) with 30 μl of saline containing 5 × 10^8^ CFU. At 4 h postinfection, the following treatments were administered once daily for 3 days: (i) exebacase (5 mg/kg i.v.) alone, (ii) daptomycin (50 mg/kg s.c.) alone, exebacase (5 mg/kg i.v.) in addition to daptomycin (50 mg/kg s.c.), and (iv) sterile saline (vehicle control). Ten animals were included for each group. Mortality was monitored over a 14-day period. For each treatment group, sets of 4 mice were set aside for CFU determinations; lung tissue samples were collected, suspended in filter-sterilized PBS, and homogenized with a tissue homogenizer. Quantitative plating was then performed to measure the bacterial loads in the lungs. This study was performed under the guidelines and protocols of the Institutional Animal Care and Use Committee of ContraFect Corporation.

## References

[1] Sakr A, Bregeon F, Mege JL, Rolain JM, Blin O. 2018. *Staphylococcus aureus* nasal colonization: an update on mechanisms, epidemiology, risk factors, and subsequent infections. Front Microbiol 9:2419. 10.3389/fmicb.2018.02419. 30349525 PMC6186810

[2] Wertheim HF, Melles DC, Vos MC, van Leeuwen W, van Belkum A, Verbrugh HA, Nouwen JL. 2005. The role of nasal carriage in *Staphylococcus aureus* infections. Lancet Infect Dis 5:751–762. 10.1016/S1473-3099(05)70295-4. 16310147

[3] Tong SY, Davis JS, Eichenberger E, Holland TL, Fowler VG, Jr. 2015. *Staphylococcus aureus* infections: epidemiology, pathophysiology, clinical manifestations, and management. Clin Microbiol Rev 28:603–661. 10.1128/CMR.00134-14. 26016486 PMC4451395

[4] Wuerth BA, Bonnewell JP, Wiemken TL, Arnold FW. 2016. Trends in pneumonia mortality rates and hospitalizations by organism, United States, 2002-2011. Emerg Infect Dis 22:1624–1627. 10.3201/eid2209.150680. 27532154 PMC4994371

[5] Morris DE, Cleary DW, Clarke SC. 2017. Secondary bacterial infections associated with influenza pandemics. Front Microbiol 8:1041. 10.3389/fmicb.2017.01041. 28690590 PMC5481322

[6] Morens DM, Taubenberger JK, Fauci AS. 2008. Predominant role of bacterial pneumonia as a cause of death in pandemic influenza: implications for pandemic influenza preparedness. J Infect Dis 198:962–970. 10.1086/591708. 18710327 PMC2599911

[7] Vaillancourt M, Jorth P. 2020. The unrecognized threat of secondary bacterial infections with COVID-19. mBio 11:e01806-20. 10.1128/mBio.01806-20. 32769090 PMC7419722

[8] Zhou F, Yu T, Du R, Fan G, Liu Y, Liu Z, Xiang J, Wang Y, Song B, Gu X, Guan L, Wei Y, Li H, Wu X, Xu J, Tu S, Zhang Y, Chen H, Cao B. 2020. Clinical course and risk factors for mortality of adult inpatients with COVID-19 in Wuhan, China: a retrospective cohort study. Lancet 395:1054–1062. 10.1016/S0140-6736(20)30566-3. 32171076 PMC7270627

[9] Centers for Disease Control and Prevention. 2019. Antibiotic resistance threats in the United States. Centers for Disease Control and Prevention, US Department of Health and Human Services, Atlanta, GA.

[10] World Health Organization. 2020. Antibiotic resistance. World Health Organization, Geneva, Switzerland. https://www.who.int/news-room/fact-sheets/detail/antibiotic-resistance.

[11] Sehgal P, Pollanen M, Daneman N. 2019. A retrospective forensic review of unexpected infectious deaths. Open Forum Infect Dis 6:ofz081. 10.1093/ofid/ofz081. 30956993 PMC6441567

[12] Baker SJ, Payne DJ, Rappuoli R, De Gregorio E. 2018. Technologies to address antimicrobial resistance. Proc Natl Acad Sci U S A 115:12887–12895. 10.1073/pnas.1717160115. 30559181 PMC6304975

[13] Wittekind M, Schuch R. 2016. Cell wall hydrolases and antibiotics: exploiting synergy to create efficacious new antimicrobial treatments. Curr Opin Microbiol 33:18–24. 10.1016/j.mib.2016.05.006. 27257994

[14] Nelson DC, Schmelcher M, Rodriguez-Rubio L, Klumpp J, Pritchard DG, Dong S, Donovan DM. 2012. Endolysins as antimicrobials. Adv Virus Res 83:299–365. 10.1016/B978-0-12-394438-2.00007-4. 22748813

[15] Schuch R, Lee HM, Schneider BC, Sauve KL, Law C, Khan BK, Rotolo JA, Horiuchi Y, Couto DE, Raz A, Fischetti VA, Huang DB, Nowinski RC, Wittekind M. 2014. Combination therapy with lysin CF-301 and antibiotic is superior to antibiotic alone for treating methicillin-resistant *Staphylococcus aureus*-induced murine bacteremia. J Infect Dis 209:1469–1478. 10.1093/infdis/jit637. 24286983 PMC3982849

[16] Watson A, Sauve K, Cassino C, Schuch R. 2020. Exebacase demonstrates *in vitro* synergy with a broad range of antibiotics against both methicillin-resistant and methicillin-susceptible *Staphylococcus aureus*. Antimicrob Agents Chemother 64:e01885-19. 10.1128/AAC.01885-19. 31712212 PMC6985718

[17] Watson A, Oh JT, Sauve K, Bradford PA, Cassino C, Schuch R. 2019. Antimicrobial activity of exebacase (lysin CF-301) against the most common causes of infective endocarditis. Antimicrob Agents Chemother 63:e01078-19. 10.1128/AAC.01078-19. 31332073 PMC6761524

[18] Schuch R, Khan BK, Raz A, Rotolo JA, Wittekind M. 2017. Bacteriophage lysin CF-301, a potent antistaphylococcal biofilm agent. Antimicrob Agents Chemother 61:e02666-16. 10.1128/AAC.02666-16. 28461319 PMC5487678

[19] Oh JT, Cassino C, Schuch R. 2019. Postantibiotic and sub-MIC effects of exebacase (lysin CF-301) enhance antimicrobial activity against *Staphylococcus aureus*. Antimicrob Agents Chemother 63:e02616-18. 10.1128/AAC.02616-18. 30936103 PMC6535547

[20] Oh J, Sauve K, Watson A, Jandourek A, Abdelhady W, Xiong YQ, Bayer AS, Cassino C, Schuch R. 2018. Lysin CF-301 (exebacase) resensitizes methicillin-resistant *Staphylococcus aureus* (MRSA) to penicillin derivatives and first generation cephalosporins. Abstr 2018 ESCMID/ASM Conf Drug Dev Meet Chall Antimicrob Resist, Lisbon, Portugal.

[21] Fowler VG, Jr, Das AF, Lipka-Diamond J, Schuch R, Pomerantz R, Jauregui-Peredo L, Bressler A, Evans D, Moran GJ, Rupp ME, Wise R, Corey GR, Zervos M, Douglas PS, Cassino C. 2020. Exebacase for patients with *Staphylococcus aureus* bloodstream infection and endocarditis. J Clin Invest 130:3750–3760. 10.1172/JCI136577. 32271718 PMC7324170

[22] ClinicalTrials.gov. 2019. Direct Lysis of Staph Aureus Resistant Pathogen Trial of Exebacase (DISRUPT). https://clinicaltrials.gov/ct2/show/NCT04160468?term=contrafect&draw=2&rank=1.

[23] Silverman JA, Mortin LI, Vanpraagh AD, Li T, Alder J. 2005. Inhibition of daptomycin by pulmonary surfactant: in vitro modeling and clinical impact. J Infect Dis 191:2149–2152. 10.1086/430352. 15898002

[24] Speer CP, Sweet DG, Halliday HL. 2013. Surfactant therapy: past, present and future. Early Hum Dev 89(Suppl 1):S22–S24. 10.1016/S0378-3782(13)70008-2. 23809343

[25] Clinical and Laboratory Standards Institute. 2020. Performance standards for antimicrobial susceptibility testing, document M100, 30th ed. Clinical and Laboratory Standards Institute, Wayne, PA.

[26] Bergstrom F, Mikhalyov I, Hagglof P, Wortmann R, Ny T, Johansson LB. 2002. Dimers of dipyrrometheneboron difluoride (BODIPY) with light spectroscopic applications in chemistry and biology. J Am Chem Soc 124:196–204. 10.1021/ja010983f. 11782171

[27] Moody J. 2010. Synergy testing: broth microdilution checkerboard and broth macrodilution methods, p 5.12.1–5.12.23. *In* Garcia LS (ed), Clinical microbiology procedures handbook, 3rd ed and 2007 update, vol 2. ASM Press, Washington, DC.

[28] Sakoulas G, Rose W, Berti A, Olson J, Munguia J, Nonejuie P, Sakoulas E, Rybak MJ, Pogliano J, Nizet V. 2017. Classical beta-lactamase inhibitors potentiate the activity of daptomycin against methicillin-resistant Staphylococcus aureus and colistin against Acinetobacter baumannii. Antimicrob Agents Chemother 61:e01745-16. 10.1128/AAC.01745-16. 27872080 PMC5278754

[29] Hall Snyder AD, Werth BJ, Nonejuie P, McRoberts JP, Pogliano J, Sakoulas G, Yim J, Singh N, Rybak MJ. 2016. Fosfomycin enhances the activity of daptomycin against vancomycin-resistant enterococci in an in vitro pharmacokinetic-pharmacodynamic model. Antimicrob Agents Chemother 60:5716–5723. 10.1128/AAC.00687-16. 27431211 PMC5038233

[30] Grein F, Muller A, Scherer KM, Liu X, Ludwig KC, Klockner A, Strach M, Sahl HG, Kubitscheck U, Schneider T. 2020. Ca(2+)-daptomycin targets cell wall biosynthesis by forming a tripartite complex with undecaprenyl-coupled intermediates and membrane lipids. Nat Commun 11:1455. 10.1038/s41467-020-15257-1. 32193379 PMC7081307

[31] Indiani C, Sauve K, Raz A, Abdelhady W, Xiong YQ, Cassino C, Bayer AS, Schuch R. 2019. The antistaphylococcal lysin, CF-301, activates key host factors in human blood to potentiate methicillin-resistant *Staphylococcus aureus* bacteriolysis. Antimicrob Agents Chemother 63:e02291-18. 10.1128/AAC.02291-18. 30670427 PMC6437495

[32] Schwameis R, Erdogan-Yildirim Z, Manafi M, Zeitlinger MA, Strommer S, Sauermann R. 2013. Effect of pulmonary surfactant on antimicrobial activity in vitro. Antimicrob Agents Chemother 57:5151–5154. 10.1128/AAC.00778-13. 23877678 PMC3811439

[33] Liu C, Bayer A, Cosgrove SE, Daum RS, Fridkin SK, Gorwitz RJ, Kaplan SL, Karchmer AW, Levine DP, Murray BE, Rybak MJ, Talan DA, Chambers HF. 2011. Clinical practice guidelines by the Infectious Diseases Society of America for the treatment of methicillin-resistant Staphylococcus aureus infections in adults and children: executive summary. Clin Infect Dis 52:285–292. 10.1093/cid/cir034. 21217178

[34] Clinical and Laboratory Standards Institute. 2018. Methods for dilution antimicrobial susceptibility tests for bacteria that grow aerobically, document M07, 11th ed. Clinical and Laboratory Standards Institute, Wayne, PA.

[35] Clinical and Laboratory Standards Institute. 2017. AST Subcommittee Working Group meetings and plenary (Jan. 16-17). Clinical and Laboratory Standards Institute, Wayne, PA. https://clsi.org/meetings/ast-file-resources/.

[36] Sá-Leão R, Santos Sanches I, Dias D, Peres I, Barros RM, de Lencastre H. 1999. Detection of an archaic clone of *Staphylococcus aureus* with low-level resistance to methicillin in a pediatric hospital in Portugal and in international samples: relics of a formerly widely disseminated strain?. J Clin Microbiol 37:1913–1920. 10.1128/JCM.37.6.1913-1920.1999. 10325346 PMC84983

