# Supplementary material for: Exebacase Is Active In Vitro in Pulmonary Surfactant and Is Efficacious Alone and Synergistic with Daptomycin in a Mouse Model of Lethal Staphylococcus aureus Lung Infection
Source: Antimicrob Agents Chemother. 2021 Aug 17;65(9):e02723-20. doi: 10.1128/AAC.02723-20 (PMC8370210; doi:10.1128/AAC.02723-20)
Supplement: Supplemental file 1 — Supplemental Table S1. Download AAC.02723-20-s0001.pdf, PDF file, 0.1 MB [file aac.02723-20-s0001.pdf]

Supplemental Material

TABLE S1 Strains used in this study

Designation	Type	Description	Source and/or Reference
ATCC BAA-42	MRSA	Isolated from a child in a hospital pneumology ward, Lisbon, Portugal, 1996	ATCC (1)
ATCC BAA-1747	MRSA	Human clinical isolate	ATCC (2)
ATCC BAA-1688	MRSA	Human clinical isolate	ATCC (3)
MW2	MRSA	NRS 123, SCC*mec* type IV, PVL-positive, PFGE type USA400	BEI Resources (4)
NRS 265	MRSA	Human clinical isolate	BEI Resources
NRS 193	MRSA	Isolated from a 13-year-old female with necrotizing pneumonia and severe sepsis	BEI Resources (5)
NRS 255	MRSA	Human isolate	BEI Resources
JMI 947	MRSA	Respiratory tract infection isolate	JMI Laboratories
JMI 3167	MRSA	Respiratory tract infection isolate	JMI Laboratories
ATCC 25923	MSSA	Laboratory strain	ATCC
ATCC 29213	MSSA	Quality control strain for standard antimicrobial susceptibility testing	ATCC
ATCC 49521	MSSA		
NRS 153	MSSA	Isolated from a patient with staphylococcal scalded skin syndrome	BEI Resources (6)
NRS 131	MSSA	Human clinical isolate	BEI Resources (7)
NRS 106	MSSA	Human clinical isolate	BEI Resources (8)
JMI 316	MSSA	Respiratory tract infection isolate	JMI Laboratories
JMI 1040	MSSA	Respiratory tract infection isolate	JMI Laboratories

JMI 1173	MSSA	Respiratory tract infection isoalte	JMI Laboratories

Abbreviations = MSSA, methicillin-sensitive *S. aureus*; MRSA, methicillin-resistant *S. aureus*; PFGE, Pulsed Field Gel Electrophoresis; PVL, Panton–Valentine Leucocidin; ATCC, American Type Culture Collection; and SCC, *Staphylococcus* Cassette Chromosome.

REFERENCES

1. Sa-Leao R, Santos Sanches I, Dias D, Peres I, Barros RM, de Lencastre H. 1999. Detection of an archaic clone of *Staphylococcus aureus* with low-level resistance to methicillin in a pediatric hospital in Portugal and in international samples: relics of a formerly widely disseminated strain? J Clin Microbiol 37:1913-20.
2. Lindenmayer JM, Schoenfeld S, O'Grady R, Carney JK. 1998. Methicillin-resistant *Staphylococcus aureus* in a high school wrestling team and the surrounding community. Arch Intern Med 158:895-9.
3. Davis SL, Perri MB, Donabedian SM, Manierski C, Singh A, Vager D, Haque NZ, Speirs K, Muder RR, Robinson-Dunn B, Hayden MK, Zervos MJ. 2007. Epidemiology and outcomes of community-associated methicillin-resistant *Staphylococcus aureus* infection. J Clin Microbiol 45:1705-11.
4. Baba T, Bae T, Schneewind O, Takeuchi F, Hiramatsu K. 2008. Genome sequence of *Staphylococcus aureus* strain Newman and comparative analysis of staphylococcal genomes: polymorphism and evolution of two major pathogenicity islands. J Bacteriol 190:300-10.
5. Centers for Disease C, Prevention. 1999. Four pediatric deaths from community-acquired methicillin-resistant *Staphylococcus aureus* - Minnesota and North Dakota, 1997-1999. MMWR Morb Mortal Wkly Rep 48:707-10.
6. Jarraud S, Lyon GJ, Figueiredo AM, Lina G, Vandenesch F, Etienne J, Muir TW, Novick RP. 2000. Exfoliatin-producing strains define a fourth agr specificity group in *Staphylococcus aureus*. J Bacteriol 182:6517-22.
7. Gros MF, te Riele H, Ehrlich SD. 1987. Rolling circle replication of single-stranded DNA plasmid pC194. EMBO J 6:3863-9.
8. Archer GL, Coughter JP, Johnston JL. 1986. Plasmid-encoded trimethoprim resistance in staphylococci. Antimicrob Agents Chemother 29:733-40.
